# TRAIL-expressing mesenchymal stem cells kill the putative cancer stem cell population

**DOI:** 10.1038/sj.bjc.6605952

**Published:** 2010-11-09

**Authors:** M R Loebinger, E K Sage, D Davies, S M Janes

**Affiliations:** 1 Centre For Respiratory Research, Rayne Institute, University College London, 5 University Street, London WC1E 6JJ, UK; 2 Flow Cytometry Laboratory, Cancer Research UK, London Research Institute, 44 Lincoln's Inn Fields, London WC2A 3PX, UK

**Keywords:** mesenchymal stem cell, cancer stem cell, side population, lung cancer, TRAIL, apoptosis

## Abstract

**Background:**

Tumours contain stem-like, side population (SP) cells, which have increased tumorigenic potential, resistance to traditional therapies and may be responsible for treatment failures and relapse in patients.

**Methods:**

Mesenchymal stem cells (MSCs) were engineered to express the apoptotic ligand, TNF-related apoptosis-inducing ligand (TRAIL). Squamous (H357) and lung (A549) cancer cell lines were sorted into side and non-side populations (non-SP) by Hoechst flow cytometry. The survival and growth of both SP and non-SP cancer populations, in conjunction with TRAIL-expressing MSCs and mitoxantrone chemotherapy, were assessed by flow cytometry and colony forming ability.

**Results:**

Mesenchymal stem cells expressing TRAIL migrate to tumours and reduce the growth of primary cancers and metastases. This report demonstrates that these cells cause apoptosis, death and reduced colony formation of the SP of squamous and adenocarcinoma lung cancer cells and are synergistic when combined with traditional chemotherapy in apoptosis induction.

**Conclusions:**

The sensitivity of putative cancer stem cells to TRAIL-expressing MSCs, suggests their possible role in the prevention of cancer relapse.

Cancers are composed of a heterogeneous mix of cells with varying differentiation, proliferation and tumorigenic properties (Heppner, 1984; Loebinger *et al*, 2008b). Indeed, *in vivo* studies have demonstrated that within a cancer population, only a small percentage of potential ‘cancer stem cells’ are able to initiate tumour development (Lapidot *et al*, 1994; Al-Hajj *et al*, 2003; Loebinger *et al*, 2008b) (Visvader and Lindeman, 2008). Conventional cancer treatments may eradicate the tumour bulk but spare these cells, which may explain why an initial tumour regression does not necessarily translate to an improved patient survival in many clinical trials for advanced cancers (Wicha *et al*, 2006).

The identification and destruction of these stem cells may therefore improve cancer treatment responses. Cell surface markers have been used in some cancers to produce a population of cells enriched with stem cell properties, for example, CD133 in the identification of human glioma (Singh *et al*, 2004) and colon cancer (Ricci-Vitiani *et al*, 2007) stem cells. However these markers appear specific to particular tumours and no marker has identified cancer stem cells across tumour types. Normal stem cell characteristics are often utilised to identify this population. This includes the ability to efflux nuclear dyes such as Hoechst 33342, which binds to DNA. The efflux of Hoechst is due to ABC transporters, in particular ABCG2/BCRP1 (Zhou *et al*, 2001). These Hoechst-effluxing cells were originally described in the bone marrow and termed ‘side population’ (SP) owing to their appearance on flow cytometry plots (Goodell *et al*, 1996). They have since been shown in a variety of normal tissues, wherein they possess stem-like properties (Wu and Alman, 2008). They have also been identified in many cancers, including lung (Ho *et al*, 2007; Sung *et al*, 2008), breast (Engelmann *et al*, 2008), oesophageal (Huang *et al*, 2009), hepatocellular (Kamohara *et al*, 2008), glioma (Harris *et al*, 2008), renal (Addla *et al*, 2008) and squamous (Loebinger *et al*, 2008b) cancer cell lines, in addition to primary cancer cells (Barrett *et al*, 1995; Hirschmann-Jax *et al*, 2004; Szotek *et al*, 2006; Wu *et al*, 2007).

We, and others, have previously demonstrated that this Hoechst-effluxing, SP of cells within cancers have many stem-like properties, including the ability to re-populate both the SP and non-Hoechst efflux ability non-side populations (non-SP) cell compartments, an increased ability to form colonies and generate complex spheroids in three-dimensional culture, a high telomerase activity and increased quiescence (Kondo *et al*, 2004; Ho *et al*, 2007; Addla *et al*, 2008; Loebinger *et al*, 2008b; Zhang *et al*, 2009). They have also been shown to express a number of stem-like genes (including *OCT-4, SOX-2* and *BMI-1*) (Huang *et al*, 2009; Zhang *et al*, 2009), ABC transporter genes (including *ABCG2*) (Loebinger *et al*, 2008b; Huang *et al*, 2009; Zhang *et al*, 2009), and genes involved in the Wnt (Haraguchi *et al*, 2006; Addla *et al*, 2008; Huang *et al*, 2009), Notch (Addla *et al*, 2008; Huang *et al*, 2009), PI3K/Akt pathways and cell cycle regulation (Zhou *et al*, 2007). Side population cells derived from both primary tumours (Wu *et al*, 2007) and cancer cell lines (Chiba *et al*, 2006; Ho *et al*, 2007; Loebinger *et al*, 2008b) also have an increased ability to initiate tumours compared with the majority of the tumour cells, when xenografted into immunodeficient mice. Furthermore, we, and others, have demonstrated that the SP cells are able to escape death by many chemotherapeutic agents, owing in part to their relative quiescence, in addition to increased ABC transporter expression which leads to the efflux of lipophilic chemotherapy agents such as mitoxantrone (Hirschmann-Jax *et al*, 2004; Loebinger *et al*, 2008b). This combination of resistance and tumour initiation makes it likely that these SP cells are central to tumour growth and recurrence, and stresses the importance of targeting these cells with future cancer therapies.

Mesenchymal stem cells (MSCs) have been used as delivery vehicles for targeted, antitumour therapies (Studeny *et al*, 2002; Loebinger *et al*, 2008a, 2008c). These cells are derived from the adult bone marrow and have the ability to specifically home towards tumours throughout the body. In addition, they are immunoprivileged, enabling their use without rejection or immunosuppressive pre-conditioning. We, and others, have engineered MSCs to express TNF-related apoptosis-inducing ligand (TRAIL) (Loebinger *et al*, 2009; Szegezdi *et al*, 2009; Grisendi *et al*, 2010). TRAIL is a protein which causes apoptosis and death of cancer cells, without harming normal cells, by binding to specific TRAIL receptors and leading to activation of the extrinsic apoptosis pathway (Wiley *et al*, 1995). *In vivo* studies have demonstrated that these cells are able to target multiple tumours and reduce primary and metastatic disease (Loebinger *et al*, 2009; Grisendi *et al*, 2010). The ability of this therapy to target and kill the putative cancer stem cells has not been determined. We hypothesised that MSC-delivered TRAIL therapy would target SP and non-SP cells equally.

## Materials and methods

### Tissue culture

Human adult MSCs were provided through the Tulane Centre for Gene Therapy, MSC cell distribution centre (New Orleans, LA, USA) and cultured in *α*MEM with 4 mM L-Glutamine, 50 U ml^−1^ penicillin and 50 *μ*g ml^−1^ streptomycin, and 16% (v/v) fetal bovine serum. H357 cells were provided by Cancer Research UK, and cultured in a 1 : 3 mix of Hams F12 medium and Dulbecco's modified Eagle's medium (Janes and Watt, 2004). In addition to fetal bovine serum, L-Glutamine and antibiotics, this medium was supplemented with 10^−10^M cholera enterotoxin (ICN Pharmaceuticals Ltd., Oxon, UK), 0.5 g ml^−1^ hydrocortisone, 10 ng ml^−1^ epidermal growth factor and 5 *μ*g ml^−1^ insulin (Janes *et al*, 2004). A549 and MDAMB231 cells were provided by Cancer Research UK and cultured in Dulbecco's modified Eagle's medium.

### Production of TRAIL-transduced MSCs

Mesenchymal stem cells were transduced with membrane-bound TRAIL and green fluorescent protein, under the control of a Tetracycline-on promoter, using a lentivirus, as previously described (Loebinger *et al*, 2009). The transduced MSCs (MSCFLT) expressed TRAIL and green fluorescent protein only on addition of 10 *μ*g ml^−1^ doxycycline (Loebinger *et al*, 2009). Human TRAIL expression was verified by ELISA (R&D Systems, Abingdon, UK) as per manufacturer's instructions.

### SP identification and sorting

**Figure 1:**
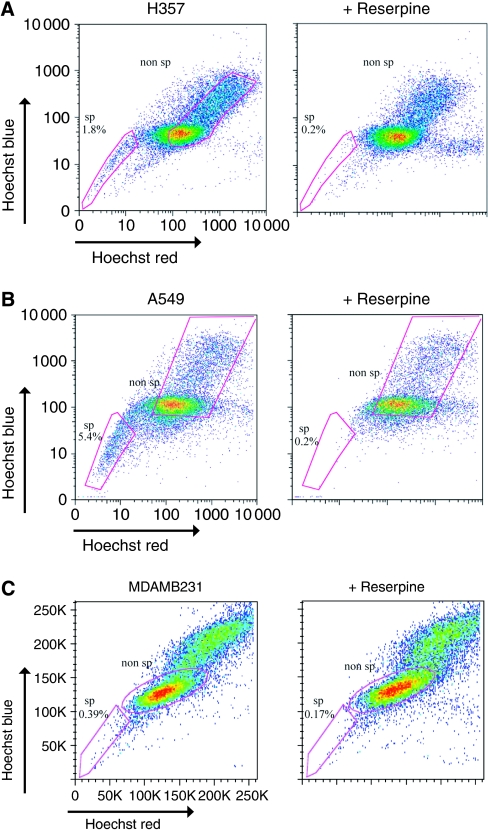
Squamous cancer and adenocarcinoma cancer cell lines contain a SP. (**A**, **B**) Representative flow cytometry plots demonstrating that H357 (**A**) and A549 cell lines (**B**) but not the MDAMB231 breast cancer cell line (**C**) contain a SP of cells that stain poorly with Hoechst. Cells were labelled with 7.5 *μ*g ml^−1^ Hoechst 33342 for 45 min. This population disappears with the ABC transporter inhibitor, reserpine (5 *μ*M). The gates show the cells defined as SP and non-SP for the experiments.

To identify the SP, previously described protocols were followed (Loebinger *et al*, 2008b). Cancer cells were harvested at full confluence, re-suspended at 1 × 10^6^ cells ml^−1^ in medium and labelled with 2.5, 5, or 7.5 *μ*g ml^−1^ Hoechst 33342 for 45, 60, or 90 min at 37°C to determine the required incubation time. Dead cells were excluded with propidium iodide labelling. The multidrug transporter inhibitor, reserpine (5 *μ*M) was used to demonstrate specificity of the SP. Analysis was performed on an LSR2 machine (Becton Dickenson, Oxford, UK). The optimal staining conditions were 5 *μ*g ml^−1^ Hoechst 33342 for 45 min and 7.5 *μ*g ml^−1^ Hoechst 33342 for 60 min for the H357 and A549 cells, respectively (Loebinger *et al*, 2008b). The SP and non-SP population (1 × 10^6^ of each cell type) were sorted according to the gates in Figure 1 using a MoFlo High-Performance Cell Sorter (Dako, Glostrup, Denmark).

### Co-culture

Cancer cells were stained with the fluorescent dye DiI (according to the manufacturer's instructions, Invitrogen, Paisley, UK), before any cell sorting, and plated with MSCFLT cells (passage seven after transduction) in a 6-well plate (5 × 10^4^ of each cell type). The following day doxycycline was added and left for 48 h. The early and late apoptosis of the cells in co-culture was assessed by Annexin V-based flow cytometry with DAPI or propidium iodide. Annexin V^−^/DAPI^−^ (or PI^−^) cells were judged to be viable, Annexin V^+^/DAPI^−^ cells were considered to be undergoing early apoptosis, and Annexin V^+^/DAPI^+^ cells were considered late apoptotic (Loebinger *et al*, 2009). In subsequent assays, the cancer cells were pre-treated with 10 ng ml^−1^ of mitoxantrone before the addition of 5 × 10^4^ MSCs (passage 9 and 10) or apoptosis induced by MSCFLT was compared with recombinant TRAIL (using manufacturer's guideline doses) for 48 h.

### Colony formation

In all, 200 DiI-stained and then freshly sorted, SP or non-SP H357 cells were added to a 6-well plate. The following day, 5 × 10^4^ MSCFLT cells, treated with mitomycin C to prevent their continued proliferation were added to the plates and the *TRAIL* transgene either activated or not with doxycycline. After 14 days of co-culture, colonies were washed, fixed using 3% PFA, and stained with Rhodanile Blue overnight. Colonies were counted using an Olympus CK2 inverted phase-contrast light microscope (Olympus, Essex, UK). A large colony was defined as greater than 32 cells per colony and abortive colonies were defined as colonies that contained fewer than 32 cells (Loebinger *et al*, 2008b).

## Results

### Squamous and lung cancer cell lines contain an ABC transporter SP

Many cancer cell lines and primary cells contain a SP. In order to identify whether squamous cell, lung and breast carcinomas contain a similar subpopulation of drug-resistant SP cells, confluent H357 (squamous), A549 (lung) and MDAMB231 (breast) cancer cell lines were incubated with Hoechst 33342 dye and analysed by flow cytometry. A characteristic SP fraction was detected in the H357 and A549 cell lines, but not in the MDAMB231 cells, which is consistent with a previous report (Engelmann *et al*, 2008). The squamous and lung SPs were both reserpine sensitive, indicating their dependence on ABC-type transporter activity (Figures 1A–C).

### The SP cells can be killed by TRAIL-expressing MSCs

**Figure 2:**
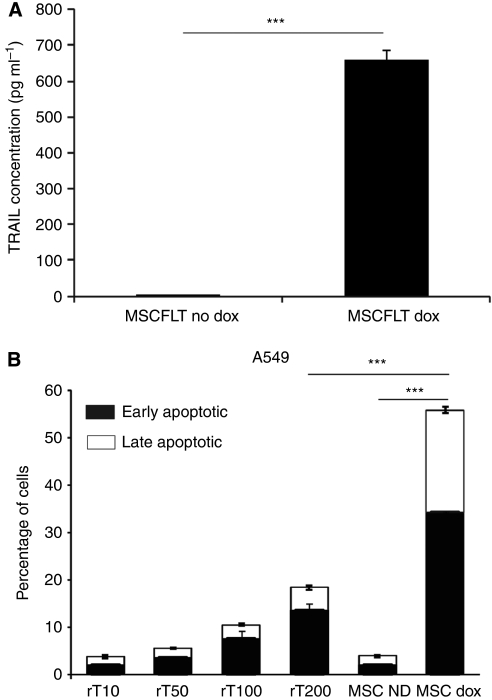
Mesenchymal stem cell TRAIL expression and killing effect compared with recombinant TRAIL. (**A**) ELISA demonstrating level of TRAIL produced in TRAIL transduced MSCs with and without addition of 10 *μ*g ml^−1^ doxycycline for 48 h. (**B**) Bar chart demonstrating the percentage of apoptosis in A549 cells with increasing levels of recombinant TRAIL (rT) 10–200 ng ml^−1^ and doxycycline-induced TRAIL expression. Experiments were performed in triplicate. ^***^P<0.001.

TRAIL-expressing MSCs have previously been shown to have the ability to cause cancer cell death and decrease tumour and metastasis development *in vivo* (Loebinger *et al*, 2009). In these experiments, the TRAIL lentivirus is conditionally activated with the addition of doxycycline, and carries green fluorescent protein to enable the monitoring of gene activation. We initially confirmed the production of TRAIL after activation of the transgene. MSCFLT cells were cultured with or without doxycyline for 48 h and cells harvested for protein measurement by ELISA. Doxycyline treatment led to 660 *vs* 2.92 pg ml^−1^ (*P*=<0.001) without treatment (Figure 2A). In co-culture experiments using H357 cells, we confirmed sensitivity of unsorted populations of these cells to doxycycline treated MSCFLT cells and found H357 cells more sensitive to MSCFLT cells than high doses of recombinant protein (Figure 2B) as previously seen by our laboratory (Loebinger *et al*, 2009).

**Figure 3:**
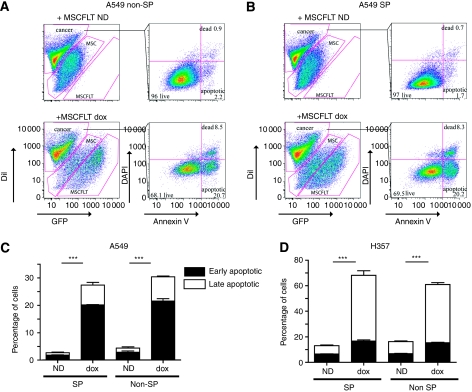
TNF-related apoptosis-inducing ligand-expressing MSCs lead to apoptosis of H357 and A549 SP and non-SP cells. Freshly sorted DiI-labelled A549 (**A**–**C**) or H357 (**D**) SP and non-SP cells were co-cultured with MSCFLT cells with (dox) or without (ND) doxycycline for 48 h. (**A**, **B**) Representative flow cytometry plots demonstrating the percentage of early and late apoptotic non-SP (**A**) and SP (**B**) A549 cells. (**C**, **D**) Bar charts representing triplicate experiments demonstrating the increase in early and late apoptosis with doxycycline-induced TRAIL expression in both the SP and non-SP subgroups of A549 (**C**) and H357 (**D**) cells. ^***^P<0.001.

The cancer stem cell hypothesis suggests that their destruction is crucial for a cancer therapy. Having isolated a population enriched for possible cancer stem cells (SP) in both squamous and lung cancer cell lines, their susceptibility to the doxycycline-controlled, TRAIL-expressing MSC therapy was tested in co-culture experiments. SP and non-SP cells were freshly flow-sorted from DiI-stained H357 and A549 cells (Figure 1) and immediately co-cultured with the MSCFLT cells. The death and apoptosis of the cancer cells in co-culture was assessed by Annexin V flow cytometry. There was a significant increase in apoptosis of both the SP and non-SP subgroups of A549 cells with the use of doxycycline and activation of the *TRAIL* transgene (SP: 2.7±0.5% increased to 27.7±1.9%, non-SP: 4.4±1.5% increased to 30.4±1.6%) (Figure 3A–C) and H357 cells (SP: 13.4±1.4% increased to 68.1±5.5%, non-SP: 16.1±1.4% increased to 60.9±2.5%) (Figure 3D) (both *P*<0.001, Anova).

**Figure 4:**
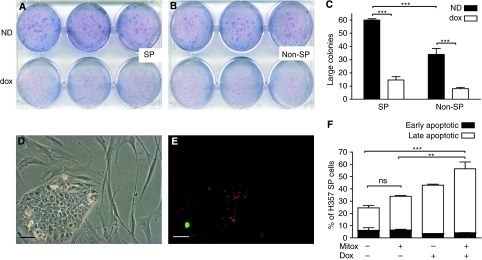
TRAIL-expressing MSCs reduce the clonogenic potential of H357 SP and non-SP cells and produce additional SP cancer cell killing to mitoxantrone treatment. (**A**, **B**) In all, 200 DiI-labelled, SP (**A**) or non-SP (**B**) H357 cells were plated for colony forming assays before the addition of 5 × 10^4^ TRAIL-expressing MSCs (MSCFLT). (**C**) Quantification of large colony numbers from (**A**, **B**) demonstrates a reduction in large colonies with doxycyline (dox)-induced TRAIL expression in both SP and non-SP cells compared with the co-cultures without dox (ND). Furthermore, SP cells produced more colonies than non-SP cells. (**D**) Phase-contrast and (**E**) fluorescent microscopy demonstrate the green fluorescent protein (green) from the doxycycline (dox)-activated MSCFLTs surrounding the DiI-labelled (red) H357 colonies. (**F**) SP cells were exposed to mitoxantrone (Mitox) and then co-cultured with MSCFLT cells. Bar chart represents triplicate flow cytometry experiments and demonstrates a further increase in death and apoptosis of the SP cells with the addition of doxycycline and activation of MSCFLT TRAIL expression. ^***^P<0.001, ^**^P<0.01, ns, non-significant. Scale bars represent 25 *μ*m.

The susceptibility of the cancer cell subpopulations to TRAIL-expressing MSCs was further assessed with colony forming assays. In all, 200 DiI-stained and then freshly sorted, SP or non-SP H357 cells were added to a 6-well plate. The following day, 5 × 10^4^ MSCFLT cells (treated with mitomycin C to prevent their further growth) were added to the plates and the *TRAIL* transgene either activated or not with doxycycline. As expected from previous work (Loebinger *et al*, 2008b), colony formation was greater in the SP subgroup compared with the non-SP cells (60.0±1.7 large colonies in the SP cells compared with 34.0±7.8 large colonies in the non-SP cells) (*P*<0.001, 2-way Anova). Colony formation was significantly inhibited in both cell subgroups with the doxycycline-induced activation of the TRAIL transgene of the MSCs (SP: 60±1.7 large colonies reduced to 14.7±4.2 large colonies with TRAIL expression, non-SP: 34.0±7.8 large colonies reduced to 8.0±1.7 large colonies with TRAIL expression) (both *P*<0.001, two-way Anova) (Figure 4A–E).

### The addition of TRAIL-expressing MSCs to mitoxantrone treatment causes further cancer cell killing

The DiI-labelled, SP H357 cells were treated with 10 ng ml^−1^ mitoxantrone for 3 days followed by co-culture with MSCFLT cells. The Annexin V flow cytometry assay was used to determine the early and late apoptotic SP cancer cells. There was a significant increase in apoptotic cells with the addition of doxycycline and activation of the *TRAIL* transgene (56.3±10.0% with doxycycline, compared with 33.9±2.4% without doxycyline) (*P*<0.01, Anova) (Figure 4F). This suggests a further cancer killing effect of the TRAIL-expressing MSCs above and beyond the mitoxantrone chemotherapy agent. Indeed, when TRAIL was not expressed with doxycycline, the use of mitoxantrone alone did not significantly increase the early and late apoptotic cancer cells, consistent with a degree of mitoxantrone chemoresistance of the SP cells as discussed above (33.9±2.4% with mitoxantrone compared with 24.56±4.2% without mitoxantrone) (*P*>0.05, Anova) (Figure 4F).

## Discussion

One feature of the SP, or subgroup of cells enriched for stem cells, is their resistance to common oncological treatments (Loebinger *et al*, 2009). These studies have demonstrated that this subpopulation has some resistance to chemotherapy agents such as mitoxantrone. This subgroup of cells has also been shown to be more capable of tumour initiation in subcutaneous models (Hirschmann-Jax *et al*, 2004; Loebinger *et al*, 2008b). The combination of increased treatment resistance and ability to re-populate tumours suggest new treatments should target these cells effectively. Our co-culture experiments with MSCFLT cells demonstrate that TRAIL-expressing MSCs are able to kill both SP and non-SP cells in squamous and adenocarcinoma lung cell lines with equal efficacy. This suggests that TRAIL-expressing MSCs could be a useful agent for cancer treatment either alone, or potentially in combination with other radiotherapy and chemotherapy regimens. The benefits of a combination approach were demonstrated in our study, with an improved SP killing when both mitoxantrone and TRAIL-expressing MSCs were used. The killing of H357 cells with activation of MSCFLT cells was higher than A549 cells (as demonstrated previously (Loebinger *et al*, 2009)) presumably due to a difference in TRAIL sensitivity.

The potential of using TRAIL to destroy putative cancer stem cells has been recognised by others. One study demonstrated TRAIL-induced apoptosis of CD133-positive glioma cells (Sasportas *et al*, 2009), a second showed radioresistant oesophageal cancer cells were 10 times more susceptible to TRAIL therapy (Zhang *et al*, 2008) and a further study noted an increased sensitivity of colon cancer SP cells to recombinant TRAIL therapy (Sussman *et al*, 2007). However, a paper by Capper *et al* showed CD133 glioma cells capable of neurosphere formation were completely resistant to TRAIL. Our study has two novel findings. First, we demonstrated that the combination of TRAIL with MSCs can target both SP and non-SP fractions and second, that their combination with traditional chemotherapies has a synergistic effect. We believe this lends important further evidence to the development of this cellular therapy in combination with current chemotherapy regimes.
